# Update on the proposed minimal standards for the use of genome data for the taxonomy of prokaryotes

**DOI:** 10.1099/ijsem.0.006300

**Published:** 2024-03-21

**Authors:** Raúl Riesco, Martha E. Trujillo

**Affiliations:** 1 Departamento de Microbiología y Genética, Campus Miguel de Unamuno, University of Salamanca, 37007 Salamanca, Spain; 2 Australian Centre for Ecogenomics, School of Chemistry and Molecular Biosciences, The University of Queensland, St Lucia, Queensland, Australia

**Keywords:** genome standards, minimal standards, genus, taxonomy, phylogenomics, OGRI

## Abstract

The field of microbial taxonomy is dynamic, aiming to provide a stable and contemporary classification system for prokaryotes. Traditionally, reliance on phenotypic characteristics limited the comprehensive understanding of microbial diversity and evolution. The introduction of molecular techniques, particularly DNA sequencing and genomics, has transformed our perception of prokaryotic diversity. In the past two decades, advancements in genome sequencing have transitioned from traditional methods to a genome-based taxonomic framework, not only to define species, but also higher taxonomic ranks. As technology and databases rapidly expand, maintaining updated standards is crucial. This work seeks to revise the 2018 guidelines for applying genome sequencing data in microbial taxonomy, adapting minimal standards and recommendations to reflect technological progress during this period.

## Data availability

Raw outputs from average amino acid identity (AAI) and percentage of conserved proteins (POCP) analyses, the R script to generate all tables, figures and statistics, and the accession codes of all the genomes used can be found at https://github.com/RiescoR/POCP-VS-AAI. Results of the AAI and POCP analyses between genome pairs are summarized in Table S3, available in the online version of this article.

## Introduction

Microbial taxonomy is an ever-evolving discipline. The accurate classification of prokaryotes not only provides insights into their evolutionary relationships and ecological roles, but also facilitates communication and collaboration across scientific disciplines. Therefore, the most important goal of microbial taxonomy is to provide a stable, objective, and up-to-date framework system of classification.

In the past, microbial taxonomy heavily relied on phenotypic characteristics, such as morphological and metabolic traits, to differentiate and classify micro-organisms. These traditional approaches, although useful, often faced limitations in providing a comprehensive understanding of microbial diversity and the underling evolution that was taking place [1,2]. The advent of molecular techniques, particularly DNA sequencing and genomics, have revolutionized our understanding of prokaryotic diversity. In the past two decades, with the advances on genome sequencing we have moved from DNA–DNA hybridization and 16S rRNA sequencing to a complete *in silico* genome-based taxonomic framework.

In a context in which technology and databases are growing exponentially and are quickly filling the gaps in diversity, it is important that we maintain an updated set of minimal standards that reflect those changes. The aim of this work is to update the general guidelines to apply genome sequencing data for taxonomic purposes released in 2018 [3], redefining some minimal standards and recommendations to reflect the technological advances during this time period.

## Use of whole genome sequence data in delineating new species

The use of overall genomic relatedness indices (OGRIs), derived from similarity or distance methods, are nowadays common in the delineation of prokaryotic species [4]. There are two commonly used indices that are usually applied to define genomic species: average nucleotide identity (ANI) and digital DNA–DNA hybridization (dDDH) [5,6]. Both have proposed (and generally accepted) thresholds that can be used for species definition (95–96 and 70 % for ANI and dDDH, respectively) [6,7]. However, while ANI and dDDH are powerful tools, it is important to acknowledge that the vast reservoir of information encapsulated within genome sequences surpasses the capabilities of conventional OGRI-based assessments. From an evolutionary perspective, ANI or dDDH cannot compete with the information about intra- or inter-species relationships contained in a genome-based phylogenetic reconstruction [8]. Additionally, the use of different tools to calculate OGRIs could also give slightly different results [9]. Thus, it is very important to analyse genomic and physiological information from several points of view and not based on a fixed threshold, especially if the compared species are very close to, or even slightly above, the thresholds.

OGRI algorithms rely to some extent on the uniformity of input data. They can be challenging to interpret for distant taxa or species with open pangenomes, those undergoing significant genome reduction, or those with a high level of horizontal gene transfer events. These circumstances could lead to a reduction in the fractions of the genome shared, which, in turn, could produce spurious results in the values, especially in metrics that do not directly incorporate this circumstance into their algorithms, such as ANI [10]. Therefore, it is important to also consider, in parallel, metrics such as the alignment fraction (AF) when using ANI or AAI indices. Multiple approaches to this problem have been proposed, including limiting the aligned genes by using higher levels of similarity [10], limiting the analysis to orthologous genes [11] or even incorporating the AF in the algorithm for evaluating taxa differentiation [12,13]. Regardless of the chosen methodology, it is important to note that OGRIs can be limited and may potentially lead to erroneous conclusions if used individually. A more balanced approach that combines multiple analyses and metrics is strongly encouraged, understanding their limitations, with the goal of providing a robust and stable framework for microbial taxonomic delineation.

**Fig. 1.**
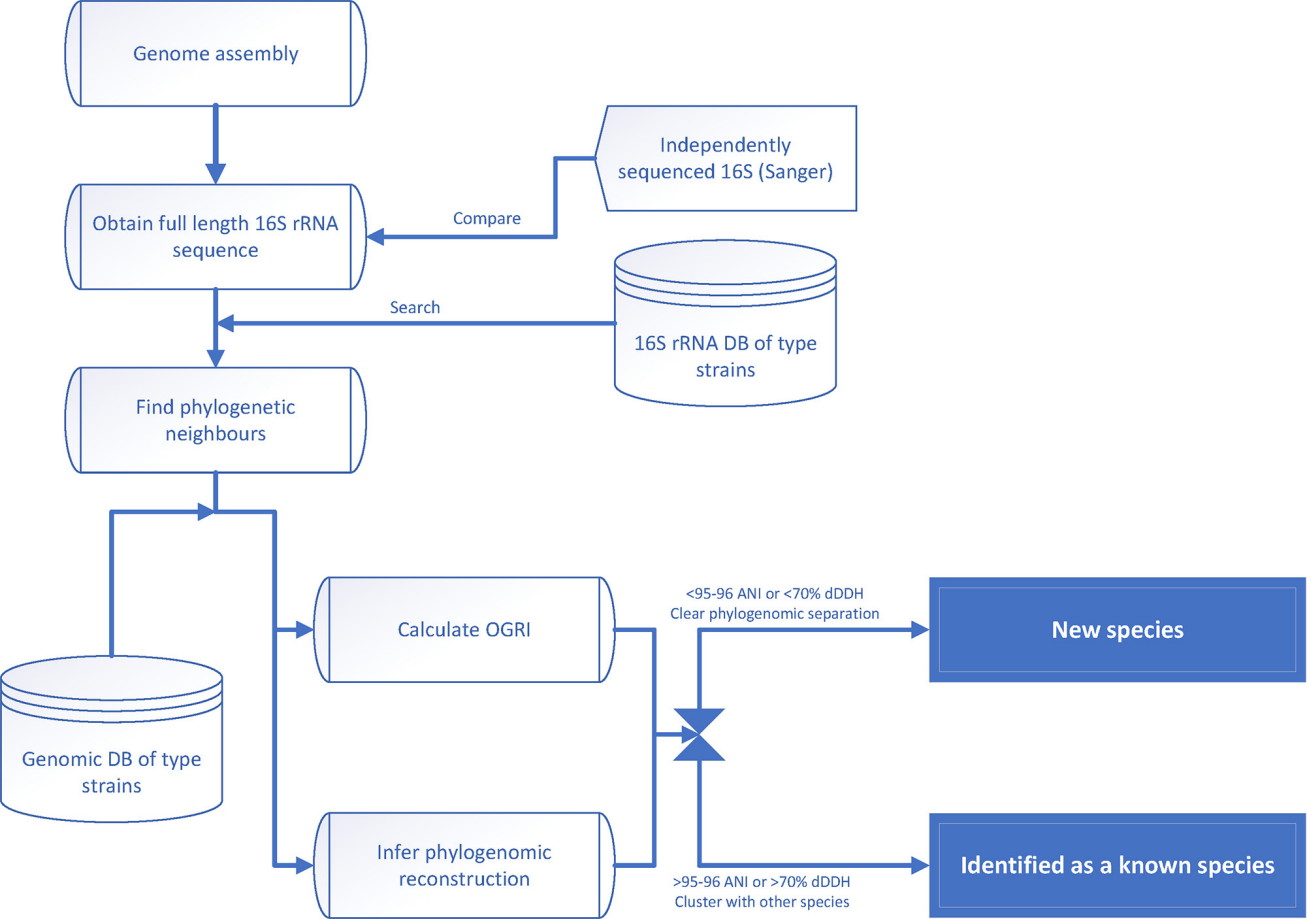
Workflow of genome-based classification at the species level.

While it is still true that not all species have a representative sequenced genome, the gap between sequenced and unsequenced type strains is steadily closing. There are even initiatives that allow the free sequencing of type material [14]. The process of genome-based comparison of species is now easier and cost-effective, even with a great number of samples involved. For that reason, the two-step process using 16S rRNA gene sequencing and OGRIs for species delineation [3] is now rarely justified. While 16S rRNA gene sequencing can still serve as a reference to infer the position of a strain at the genus or higher taxonomic level, and therefore could be used to find its taxonomic neighbours, it is recommended that phylogenetic reconstructions are made at the genomic level, placing the genome information at the centre of the analysis (Fig. 1). With the advance of genomic data, bioinformatic tools are also improving, making them more flexible, intuitive, less computer-intensive and, above all, more precise (Table 1). As one of the ultimate goals of microbial taxonomy is to devise a process of classification and identification that is stable [3], it is to be expected that, as technology and data availability fill the gaps, taxonomy ranks could ultimately be reconciled in a time-dated phylogenomic coherent classification [15].

**Table 1.** Web services and standalone software tools for taxonomic purposes

Tool	Algorithm	Function	Type	URL	Reference
ANI calculator (Kostas lab)	ANIb	Calculation of ANI	Web service	http://enve-omics.ce.gatech.edu/ani/	[27]
ANI calculator (EzBioCloud) and OrthoANIu	Orthologous ANIu	Calculation of ANI	Web service and standalone	https://www.ezbiocloud.net/tools/ani https://www.ezbiocloud.net/tools/orthoaniu	[66]
JspeciesWS	ANIb, ANIu, Tetra correlation	Calculation of ANI and Tetra-Nucleotide Analysis	Web service	https://jspecies.ribohost.com/jspeciesws/	[67]
FastANI	Mashmap/MinHash	Calculation of ANI	Standalone	https://github.com/ParBLiSS/FastANI	[68]
Genome-to-Genome Distance Calculator	GBDP	Calculation of dDDH	Web service	https://ggdc.dsmz.de/ggdc.php#	[41]
Type (Strain) Genome Server	GBDP	Calculation of dDDH, 16S phylogenetic tree reconstruction, phylogenomic tree reconstruction, genome-based classification	Web service and API	https://tygs.dsmz.de/	[40,41]
AAI calculator (Kostas lab)	blastp	Calculation of AAI	Web service	http://enve-omics.ce.gatech.edu/aai/	[27]
EzAAI	MMSeqs2	Calculation of AAI	Standalone	http://leb.snu.ac.kr/ezaai	[28]
MIGA	Pipeline with several algorithms	Assembly, calculation of ANI, AAI, genome-based classification (based on AAI or ANI)	Web service	http://microbial-genomes.org/	[69]
Global Catalogue of Type Strain (gcType) Platform	Pipeline with several algorithms	Assembly, annotation, 16S phylogenetic tree reconstruction, phylogenomic tree reconstruction, genome-based classification	Web service	https://gctype.wdcm.org/	[70]
POCP (Hoelzer)	blastp	Calculation of POCP	Standalone	https://github.com/hoelzer/pocp	[17]
POCP-matrix (Bio-py)	blastp	Calculation of POCP	Standalone	https://github.com/SilentGene/Bio-py/tree/master/POCP-matrix	[17,71]
GTDBtk	Pipeline with several algorithms	phylogenomic tree reconstruction, genome-based classification	Standalone	https://github.com/Ecogenomics/GTDBTk	[48]
PhyloPhlAn	Configurable with multiple algorithms	Phylogenomic tree reconstruction	Standalone	https://segatalab.github.io/tools/phylophlan/	[72]
IQTree	Configurable with multiple models	Maximum likelihood phylogenomic tree reconstruction from alignment	Web service and standalone	http://www.iqtree.org/	[73]
UBCG	Pipeline with several algorithms	Phylogenomic tree reconstruction of *Bacteria*	Standalone	https://www.ezbiocloud.net/tools/ubcg	[35,38]
UACG	Pipeline with several algorithms	Phylogenomic tree reconstruction of *Archaea*	Standalone	https://www.ezbiocloud.net/tools/uacg	[36]
BBMap	Multi-kmer-seed-and-extend	Calculation of sequencing depth of coverage	Standalone	https://sourceforge.net/projects/bbmap/	[74]
ContEst16S	Pipeline with several algorithms	Contamination check (16S)	Web service	https://www.ezbiocloud.net/tools/contest16s	[58]
CheckM	Pipeline with several algorithms	Calculation of completeness and contamination	Standalone	https://github.com/Ecogenomics/CheckM	[60]
CheckM2	Pipeline with several algorithms, improved with machine learning	Calculation of completeness and contamination	Standalone	https://github.com/chklovski/CheckM2	[62]
busco	Pipeline with several algorithms	Calculation of completeness and contamination	Standalone	https://busco.ezlab.org/	[61]
Kbase	Web based server with multiple programs	Server hosting multiple programs with taxonomic utility	Web service	https://www.kbase.us/	[75]

The genome also contains useful information about the ecological niche of the bacterium and could even contain significant differential markers derived from shared ecological and metabolic properties that differentiate species within a genus or a family [16]. It is therefore recommended to infer some ecological properties from the genome for the description of prokaryotic species.

## Evaluation of overall relatedness indices for the delineation of genera

While it is possible to define genera based on a combination of ANI and the alignment fraction [13], nucleotide-based OGRIs used for species delineation (ANI and dDDH) generally do not have enough resolution above the species level if we are working with a limited number of genomes [17]. Description of genera requires well resolved phylogenetic (16S or other core genes) and phylogenomic (genome-based) reconstructions based on representatives of the most related genera. These phylogenies can be complemented with OGRIs, but it is recommended to use protein-based OGRIs, instead of nucleotide-based ones. There are two protein-based indices that have gained popularity in the last few years, namely AAI and POCP [17,18]. While some guidance thresholds for genus delineation are given for both AAI (>60–65 %) [18,19] and POCP (>50 %) [17], multiple studies have proposed genus-specific boundaries; however, these values can be very different and can change from genus to genus [20]. In fact, several studies only calculate a specific boundary when describing a genus, an approach that is probably more reliable than a fixed general boundary for genus delineation, as it is usually coherent with a phylogenomic reconstruction [21,23].

To evaluate the use of POCP and AAI for genus delineation and provide a guideline for their application in the description of new genera, in this study we calculated AAI and POCP values between type strains of type species of genera within different families. To make this analysis coherent with a phylogenomic reconstruction, we used genomes with matching nomenclature at family, genus and species level in GTDB (r214) and NCBI taxonomy (release 214; see more details in Supplementary Material). The analysis included 1573 genomes and 19 874 AAI and POCP interactions, that covered relationships within 15 archaeal families (87 genera) and 197 bacterial families (1486 genera).

**Fig. 2.**
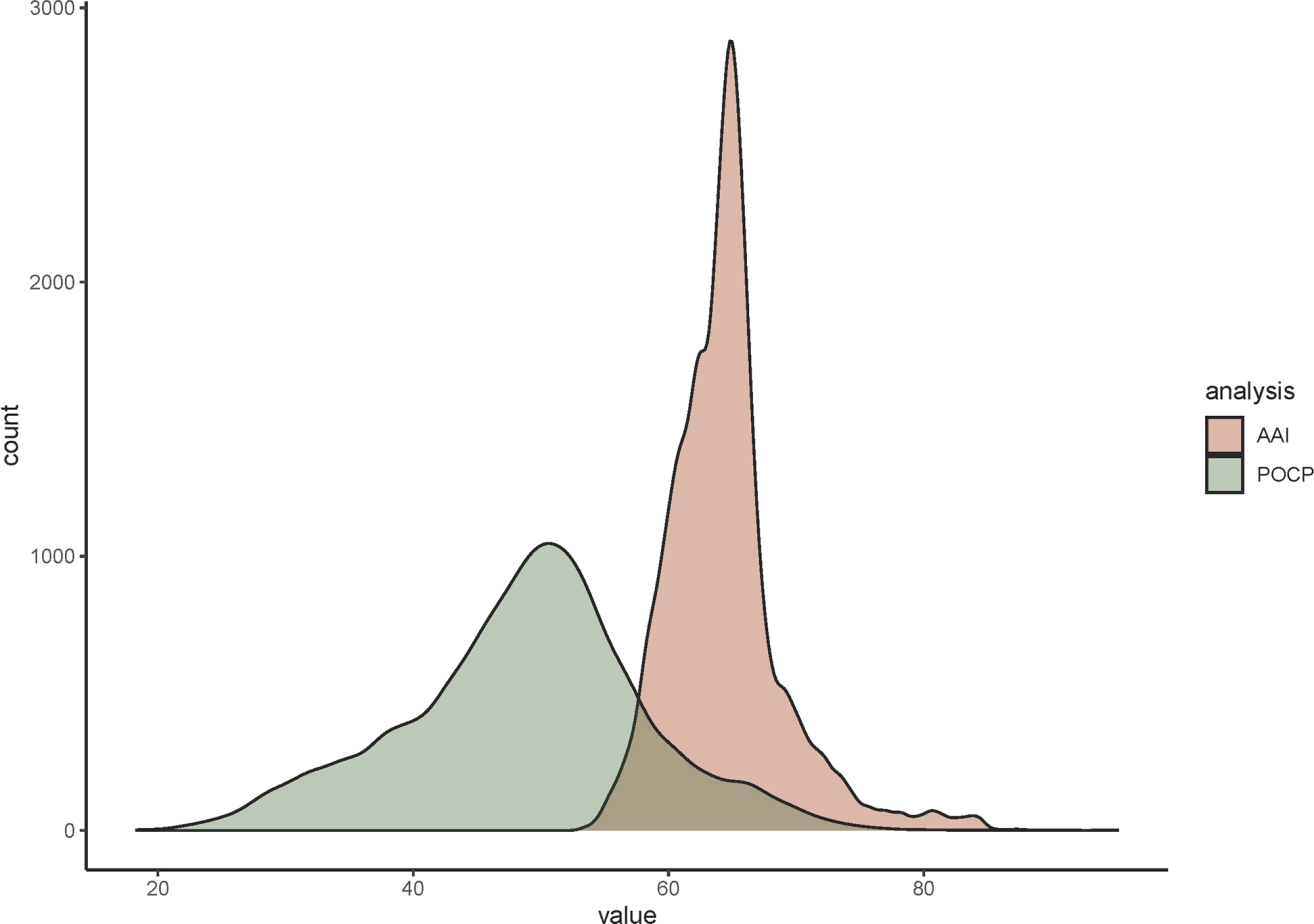
Density plot representing number of genomes for each index percentage (%). In red, AAI; in green, POPC.

**Fig. 3.**
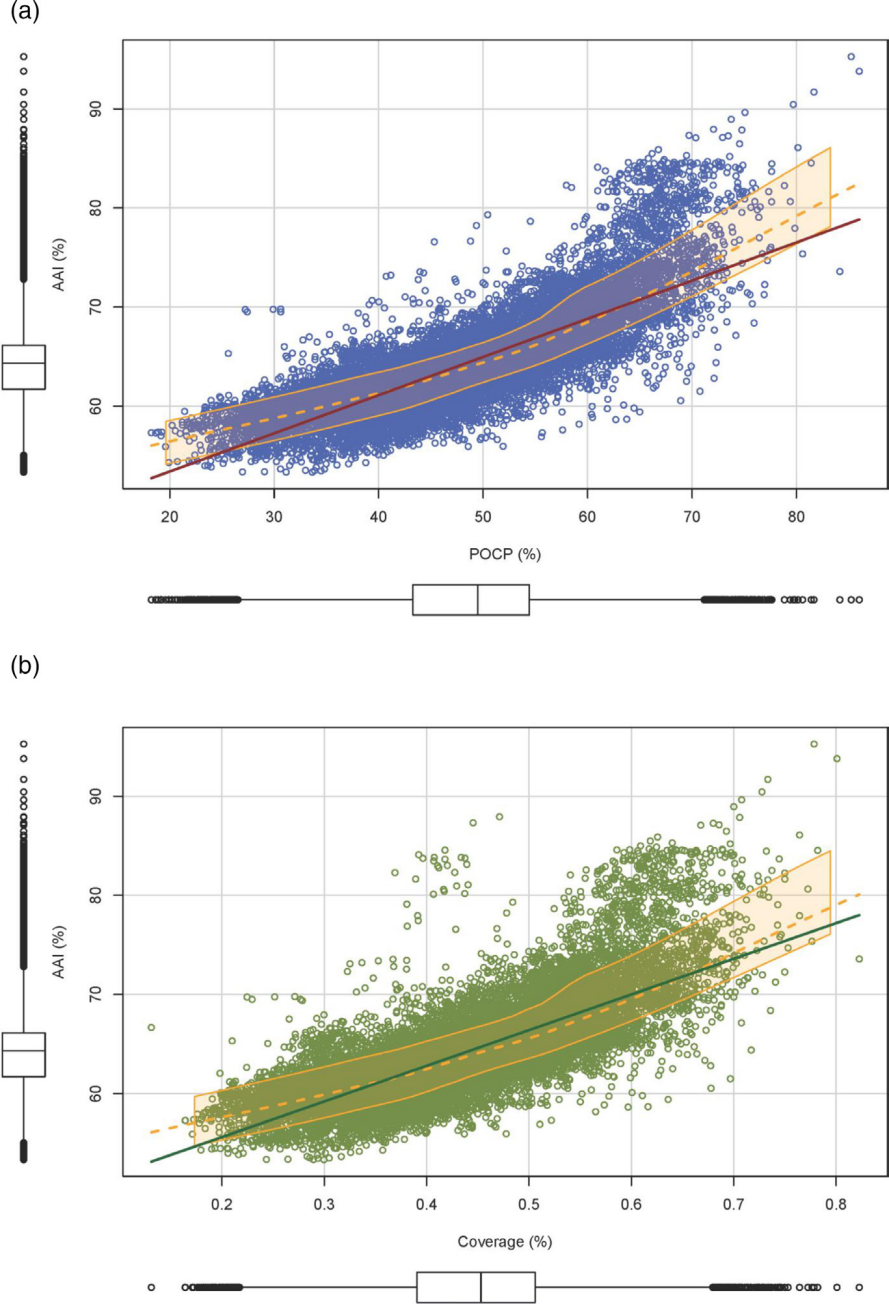
(a) AAI vs. POCP relationship. Each datapoint (blue) in the scatterplot represents a comparison between two genomes (*n*=19,874). In red, linear regression line extrapolation (R^2^=0.63); in orange, local polynomial regression and smooth derived curve. To give an idea of the distribution of the data for each variable, boxplots have been included in the graph. (**b)** AAI vs. coverage relationship. Each datapoint (green) in the scatterplot represents a comparison between two genomes (*n*=19 874). In dark green, linear regression line extrapolation (R^2^=0.56); in orange, local polynomial regression and smooth derived curve. To give an idea of the distribution of the data for each variable, boxplots have been included in the graph.

We found that AAI and POCP had a good correlation (R^2^=0.63, F stat=3.4e+04 with 19 872 degrees of freedom, *P*=2.2e-16), but AAI showed a tighter distribution than POCP (Figs2  3a) and lower standard deviation (4.5 % vs. 9.4 %). POCP distribution was symmetric, with an almost-matching mean and median, very close to the proposed genus threshold (mean=48.8 %, median=49.5 %). AAI value distribution was slightly skewed, with some outlier values on higher AAI values; however, the mean and median are almost equal (mean=64.5 %, median=64.3 %). In general, the 60 % threshold for AAI genus delineation is supported with this analysis and could even be lowered to 58 % to cover 95 % of the interactions. POCP threshold of 50 % was not appropriate, as it was only valid for 50 % of the interactions (Fig. 3a). Given the widespread distribution and deviation of POCP, a general threshold delineation for genera is not recommended. If we analyse family by family, we can appreciate that internal values of AAI and POCP varied greatly, supporting the idea that a threshold could be defined at family level, with a comprehensive analysis of the genera within the family of interest (Tables S1 and S2).

As with ANI, it is important to consider the coverage of the AAI analysis (the percentage of the proteome aligned). Low coverage and high AAI values could result in misinterpretation of these values. At the genus level, a coverage between 40–50 % is to be expected. However, this percentage could be lowered to 25 % in very low AAI values (Fig. 3b and Tables S1 and S3).

Based on these results, it is recommended the use of AAI rather than POCP to complement genome-based phylogenies at a genus level, with a minimum coverage of 25 %. While, in general, 58 % could be used as a genus threshold for AAI, the use of absolute thresholds to delineate genera is not recommended without a coherent genome-based phylogeny to support the taxonomic conclusions. In fact, the analysis of genera from a family perspective can result in very different thresholds, all of them potentially correct, ranging in AAI thresholds from 60 to 85 % for genus delineation (see Table S1), as also suggested in previous works [16,24, 25]. POCP use is not discouraged, as it correlates well with AAI, but instead it is proposed to be used as a complementary measure to AAI, given higher standard deviation. Additionally, it is important to note that POCP relatedness values are influenced by extreme differences in genome sizes [26]. Consequently, POCP should only be applied in comparisons of genomes of similar sizes.

There are several tools that allow AAI calculation, among the most popular used are the web-based AAI calculator of Kostas lab [27] and the standalone pipeline EzAAI [28]. Interestingly, the original publication of POCP did not include a tool to calculate the index [17]. However, multiple variations of this algorithm are available to the public. A list of several tools for calculation of AAI and POCP are provided in Table 1. As no comprehensive evaluation of the different pipelines has been made, it would be unwise to recommend a specific tool, therefore a set of links to a couple of GitHub repositories is given in Table 1.

As previously mentioned for species delineation, it is important to note that the exclusive use of AAI or any other OGRI parameter for genus delineation could be heavily biased by the nature of the algorithm or data input [20]. A more balanced approach that combines multiple analyses and metrics is strongly recommended, with the goal of providing a robust and stable framework for microbial taxonomic delineation.

## Use of whole genome sequence data for phylogenomic tree inference

Including genome sequences data is now usual practice when publishing taxonomic descriptions of new taxa. However, it is still common to find delineations of new species based mainly on the 16SrRNA gene sequence, using the genome data mainly to support 16S rRNA gene phylogeny by calculating OGRIs (ANI and dDDH being the most widely used). Genome-based phylogenies, or phylogenomics, are a very powerful tool that capture the complex phylogenetic relationships between different taxa, with deeper resolutions than simple OGRIs [29]. It is thus recommended that description of new taxa is centred on genome derived phylogenies.

One of the most used methodologies in phylogenomics is multigene-based phylogenies (MBPs), an evolution of the multilocus sequence analysis, in which the tree is calculated from a concatenated sequence of a wider number of orthologous genes derived from comparative genomics [30,32]. MBP is relatively easy to implement in bioinformatic pipelines in a series of well-defined steps [33] and can be derived from a fixed set of genes or even the whole core-genome [34,35]. There is no standardized minimum nor a maximum number of genes to infer an MBP; however, it has been recommended that the number of genes used should be at least 30 or above [3]. As a widely used example of this approach is the EzBioCloud UBCG pipeline, which relies on the identification of a fixed number of single copy bacterial core genes to infer a phylogeny [35]. In recent times, different versions of this pipeline have been developed, allowing for archaeal and even fungal phylogenies (UACG and UFCG) [36,38].

Another approach widely used to infer phylogenetic trees is the Genome blast Distance Phylogeny method (GBDP) [39], the underlying backbone of the Type (Strain) Genome Server (TYGS) [40,41]. Both approaches offer good and reliable phylogenies that can be used both in species and superior rank delineation [42,44].

The interpretation of phylogenetic data might not be as straightforward as it appears, because clustering does not inherently imply taxonomic separation. Phylogenies can be significantly biased by the data used, as well as the methodologies applied in alignment and reconstruction [33,45]. An appropriate distribution of data is essential. For instance, it is illogical to infer a phylogenetic tree for a genus using genomes from a different family. Moreover, selecting a suitable outgroup is crucial [46]. Factors such as the accumulation of horizontal transfer events or missing genes in the alignment, which might occur due to genome reduction or sequencing limitations, can also impact phylogenies, particularly MBP phylogenies [47]. It is always recommended to check the alignments, especially if the phylogenetic reconstruction yields conflicting data when compared to other analysis. Metrics as the gene support index, which indicate how many individual genes support a node in phylogenomic trees are useful for pinpointing these issues [35]. In phylogenomic reconstructions, where alignments are typically large, is important to note that low bootstrap values can be more significant than in single-gene phylogenetic reconstructions, while high bootstrap values can sometimes be misleading [33].

## All-in-one resources for genome-based classification of prokaryotes

All-in-one resources are pipelines that combine phylogenomic methodologies with genomic indices to allow a start-to-end classification of an assembled genome. These pipelines are highly dependent on reliable, curated, and up-to-date databases, resources that are both expensive and difficult to maintain. It is important to note that these resources are not envisioned as a substitution of a comprehensive taxonomic work, they are designed to give the user a general view of the taxonomic position of the query genome. While there are several tools available at this moment (see Table 1), there are two publicly available resources that are commonly used in taxonomy: the TYGS and the Genome Taxonomy Database toolkit (GTDBtk) [41,48].

*TYGS*: This server is a web-based resource connected to an extensive and up-to-date genomic and nomenclatural database maintained by the Leibniz Institute - Deutsche Sammlung von Mikroorganismen und Zellkulturen (DSMZ) [41]. It allows the user to infer dDDH indices and both 16S rRNA gene and GBDP-based phylogenies with the closest phylogenetic neighbours (derived from a pre-screen using Mash genomic distances and 16S rRNA gene data). It provides access to the latest nomenclature changes and related taxonomic literature, as it is connected to the same database behind LPSN [40,41, 49]. The server will also give a provisional genome-based classification at a species level, highlighting potential new species. The databases behind the service are updated and curated on a regular basis.

*GTDBtk*: The GTDB is an initiative that aims to provide a phylogenomic consistent and rank normalized taxonomy based on genomic data [50]. Species clusters in GTDB are formed using ANI (>95 %) and superior ranks are then inferred using relative evolutionary divergence indexes (RED) derived from an MBP-phylogenomic reconstruction. This approach allows a normalization of higher ranks and the inclusion of genomes reconstructed from uncultured samples. However, it is important to note that the genome-normalized taxonomy is not always consistent with formal nomenclature and name validation. GTDBtk is a standalone pipeline connected to the GTDB [48]. THe GTDBtk first places the genome in a backbone pre-calculated MBP phylogenomic tree, calculates RED indexes and then makes the species assignment using ANI, if possible. This approach allows recognition of potential new taxa at species or higher taxonomic levels. The GTDB and backbone trees are curated and updated on a regular basis, with a major update each year.

## Updated minimal standards for the use of genome sequence data for taxonomic purposes

### DNA sequencing platforms

The evolution of sequencing technologies has made whole-genome sequencing easier and accessible for all areas of biological research. A wide choice of commercial sequencing services providing adequate genome data for taxonomic purposes is available [51].

It should be stressed that the inclusion of genome sequence data in the description of prokaryotic taxa may serve other purposes. The data generated can be used to classify a micro-organism and help us infer its potential metabolism and ecological niche. In addition, the sequenced genome may serve as reference material in other fields such as clinical, environmental, and industrial microbiology. Therefore, it is very important that the end-user chooses quality over quantity at the time of selecting the sequencing technology.

At present there are several next generation sequencing (NGS) platforms that have been widely used in taxonomy and meet the quality criteria. DNA sequencing platforms provided by Illumina, Ion Torrent (Thermo Fisher Scientific), DNBSEQ (MGI) and Pacific Biosciences have proven very effective and are the most widely used and cited. These technologies can be used alone or in tandem to procure high quality genomes using the right protocols. Other sequencing platforms such as the fourth generation Oxford Nanopore, regularly used in metagenomic analysis, are steadily improving their error rate [52] and can be used to complement other platforms for genomes derived from isolates. As NGS is an ever-evolving field, it is to be expected that new technologies will be available in the future and that present technologies become even more reliable. However, new platforms should be subjected to rigorous evaluation before they can be used in taxonomic studies.

### Quality of raw data and assemblies

All NGS platforms provide their own raw data quality checks, that are comparable between each other as they all use statistics derived from the Sanger sequencing technologies [53]. Low-quality raw data is usually filtered out before the actual assembly process. The assembly and curation of the contigs is an important step, particularly in genomes derived from environmental samples instead of isolates. There are a wide variety of pipelines available, and the use of one over the other will depend on the type of data and sequencing technology used [54,55]. Although it has become customary to release only assembled genomes in public databases (GenBank, European Nucleotide Archive, etc.), the recommended approach is to upload both raw and assembled data and provide both accession numbers when publishing it. This practice is motivated by the potential divergence in outcomes from diverse assembly pipelines, which may be influenced by erroneous variable inputs or the nature of the sample. Raw data can be reassembled and even used in combination with other sequencing data if the need arises. For taxonomic purposes, it is recommended to always indicate the bioinformatic tools used, clearly specifying the version of the program to ensure reproducibility.

In prokaryotic systematics, the most relevant statistics are derived from the quality of the final assembly and not from the raw read data. While these statistics have limited use in practical analysis, they are good measures of the quality of the sequencing process and reliability of the data. The following indices are specially recommended to evaluate the quality of the genomic data for taxonomic purposes [3]:

*Assembly size*: It is defined as the sum of the length of all contigs. It is important to notice that this value only represents an approximation when the genome is not complete. It can be greatly over or underestimated in some instances. As an illustration, highly fragmented genomes frequently lead to an overestimation of genome sizes. Genome-wide associations between genome sizes and other environmental or biological parameters must always account for this bias.

*The number of contigs, N50 and L50*: As mentioned before, genome fragmentation can have many consequences, such as miscalculation of genome size or OGRI parameters. While the ideal is to have a closed genome, in most circumstances this is not possible or cost-efficient, resulting in contigs of various lengths. Very short contigs are usually excluded from the final assemblies. However, as there is not a clear standard on how to select contigs, the absolute number of contigs is not a really good quality indicator. Indices like the N50 (length of the shortest contig that accumulatively show 50 % or more of the genome size) or L50 (smallest number of contigs that sums half of genome size) give better assessment of the genome quality. Higher N50 and lower L50 will represent higher genome qualities. As a wide reference, we recommend the use of genomes with less than 1000 contigs, preferably 500 or less, and a N50 >5 kb.

*Sequencing depth of coverage*. This value determines the average number of times that each base has been read in the sequencing process. It is usually expressed in folds. As a general recommendation, a minimum sequencing depth of 50× can be used for taxonomic purposes (50× means that each base has been read 50 times on average). Nevertheless, higher values represent deeper sequencing and better sequencing results. Sometimes, especially when working with environmental samples (Metagenome-assembled genomes (MAGs), Single amplified genomes (SAGs)), a uniform depth sequencing cannot be assumed [56], so mapping the reads to the assembly is a good practice to ensure that this statistic is reliable.

*16S rRNA presence in the genome*: It is recommended that authors sequence the 16S rRNA coding gene independently from the genome (Sanger method), and then compare with the one extracted from it. This check must always be done by the authors before submitting the data to public databases, as they will be used as references for every analysis made in the future involving that species. There are examples in the literature when this check was not properly done, leading to problems in the identification of closely related species [57]. In some cases, 16S rRNA genes are present in several copies in the genome, so it is also possible to compare different copies to ensure that the genome is not contaminated [58].

*Completeness and contamination indices*: Contamination can occur in different steps of the experiment, from contamination of the isolate (not only from bacterial sources, but also viral) to the DNA sequencing process [58]. In metagenomic experiments, the binning process can also be challenging [59]. While contamination can be detected by recovering key loci in the genomes, looking for different copies of the same gene (e.g., 16S rRNA), the approach is not ideal to quantify the scale of the contamination, as these genes may not be recovered in the assembly. In the example of the 16S rRNA gene, sometimes the assembly process does not recover all (or any) of the copies in the genome, making the comparison of the copies not possible. Instead of looking for a single marker gene, it is possible to estimate both completeness and contamination in a genome by looking for the presence and identity of a collection of single-copy gene markers. There are several tools that allow to calculate these two parameters, of which CheckM and busco are the most widely used [60,61]. Recently, a new version of CheckM (CheckM2) has been published, improving its accuracy and computational speed with the introduction of machine learning in their pipeline [62]. A genome is typically considered high quality with >90 % completeness and <5 % contamination [63,64]. However, even though we should aim for the highest quality possible, for taxonomic purposes it may be sufficient to infer good-enough results with medium-quality genomes (>50 % completeness and <10 % contamination). It is important to note that while it is possible to calculate basic genomic indices with low quality genomes, we do not recommend their use as references in phylogenomic analyses. Completeness and contamination indices should be considered together, as they are closely related. As an example, the GTDB defines a ‘quality score’ index, using the formula quality score=completeness – 5*contamination, filtering out genomes that do not reach a quality score of 50 (https://gtdb.ecogenomic.org/faq). As the GTDB quality score formula implies, contamination must be heavily penalized without forgetting the completeness. Ten percent contamination automatically discards the genome for analysis, and the same could be said for 50 % completeness. While we mention >50 % completeness and <10 % contamination as absolute independent minimum values, when combined, they will result in a genome of low quality for taxonomic purposes.

### Public repositories, selection of reference data and deposition of sequencing data

Taxonomy relies on the use of updated and curated data. If genomic data is needed, members of the International Nucleotide Sequence Database Collaboration (INSDC: NCBI, ENA and DDBJ) maintain curated and stable databases that can be used to download or deposit genomic data safely and efficiently. Genomic data generated for taxonomic purposes must always be deposited in one of the databases of the INSDC, particularly assembled genomes. It is recommended that NGS raw data is also submitted to public databases, as it can be useful for the scientific community. If the end-user wants to download a reference genome, and has multiple options available, it is recommended to always download the assembly with the highest quality, attending to the quality recommendations mentioned above.

In the last few years, several databases with application in taxonomy have greatly improved and offer stable and updated repositories of taxonomy-related metadata (Table 2). Among these databases we can highlight the interconnected DSMZ web services LPSN for nomenclatural data [41,49] and BacDive for standardized strain metadata (ecology, morphology, physiology, etc.) [65]. GTDB is also a widely used metadata repository, especially in regard to metagenome-derived studies [50]. GTDB uses a fully exportable genome-based normalized taxonomy with several tools that allows its implementation in more complex bioinformatic pipelines.

**Table 2.** Databases useful for taxonomic purposes and metadata compilation

Database	Data available	URL	Reference
NCBI	Genome data, single nucleotide data, taxonomy, sample data, experiment data, reference data	https://www.ncbi.nlm.nih.gov/	[76]
ENA/EBI	Genome data, single nucleotide data, taxonomy, sample data, experiment data, reference data	https://www.ebi.ac.uk/ena	[77]
DDBJ	Genome data, single nucleotide data, taxonomy, sample data, experiment data, reference data	https://www.ddbj.nig.ac.jp/	[78]
JGI	Genome data (limited), sample data, experiment data	https://genome.jgi.doe.gov/portal/	[79]
Ensembl Genomes	Genome data	https://bacteria.ensembl.org/	[80]
LPSN	Taxonomy, nomenclature, 16s data, reference data	https://www.bacterio.net/	[49]
EzBioCloud	Taxonomy, genome data, 16S data	https://www.ezbiocloud.net/	[81]
BacDive	Strain metadata (physiology, morphology, ecology, culture media, etc.)	https://bacdive.dsmz.de/	[65]
GTDB	Taxonomy, genome metadata	https://gtdb.ecogenomic.org/	[50]

## Conclusions

We recommend the following to use a genome sequence for taxonomic purposes in prokaryotes:

- Extraction of genomic material, library construction, sequencing platform and assembly methodology should always be described in the methodology. For reproducibility, versions of all bioinformatic tools used must always be specified and referenced.
- The following statistics must always be defined with the final assembly: 1) genome size; 2) DNA G+C ratio; 3) number of contigs; 4) N50 and/or L50, 5) sequencing depth; 6) completeness estimation; 7) contamination estimation. We recommend the use of genomes with a minimum of 50× depth coverage, fewer than 1000 contigs, N50 >5 kb, >50 % completeness and <10 % contamination.
- To ensure the authenticity of the genome, the 16S rRNA gene sequence must be sequenced independently from the genome (Sanger) and compared with the sequence extracted from the genome.
- For the proposal of new species, OGRI values and phylogenomic trees should be used as central references in the analysis. OGRI thresholds must be used as a reference and not as absolute values. The use of all data and, especially, phylogenomic reconstruction, can justify separation of species above the recommended thresholds. Inferring metabolic and ecological proprieties from the genome or differential genomic markers is also advisable.
- For the proposal of new genera, a combination of protein-based OGRI and phylogenomic reconstruction should be the preferred methodology.
- Genome assembly must be deposited in public databases with no access restrictions. Deposition of raw NGS data is also recommended.
- If multiple reference genomes are available in public databases, it is recommended to choose the reference genome assembly with the highest quality among the available options for its use in taxonomy.

## supplementary material

10.1099/ijsem.0.006300 Supplementary Material 1.

10.1099/ijsem.0.006300 Table S1.
