# Supplementary material for: Update on the proposed minimal standards for the use of genome data for the taxonomy of prokaryotes
Source: Int J Syst Evol Microbiol. 2024 Mar 21;74(3):006300. doi: 10.1099/ijsem.0.006300 (PMC10963913; doi:10.1099/ijsem.0.006300)
Supplement: Supplementary Material 1. [file ijsem-74-06300-s001.pdf]

Update on the proposed minimal standards for the use of genome data for the taxonomy of prokaryotes

Raúl Riesco[1*,2], Martha E. Trujillo[1]

[1]: Departamento de Microbiología y Genética, Campus Miguel de Unamuno, University of Salamanca, 37007 Salamanca, Spain.

[2]: Australian Centre for Ecogenomics, School of Chemistry and Molecular Biosciences, The University of Queensland, St Lucia, Queensland, Australia.

*Corresponding author: Raúl Riesco shot89_1000@usal.es / raul.riescoj@usal.es

Supplementary material 1: Evaluation of overall relatedness indices in the delineation of genera, detailed material and methods.

GTDB metadata databases (release 214) for Archaea and Bacteria were downloaded from GTDB (https://data.gtdb.ecogenomic.org/releases/latest/). Selection of genomes was made in R, using the following rules:

- Genomes belonging to the type strain of the type species of genus. In this analysis we compare only one genome (the type strain of the type species) within genera of the same family.
- Genomes that were "representative genomes" in GTDB database.
- Genomes that shared identical taxonomy nomenclature at the family, genus, and species levels in both GTDB genome-based taxonomy (release r214) and NCBI taxonomy (release 214) were identified. This step was crucial, as we aimed to adhere to a normalized phylogenomic coherent classification and ICSP formal nomenclature, while avoiding the 'lumping' or 'slicing' of taxa below the family level.
- Genomes in families with only one representative were discarded.

This selection resulted in 1573 genomes, 87 archaeal genera representatives (4 phyla, 10 classes, 10 orders, 15 families) and 1486 bacterial genera representatives (28 phyla, 46 classes, 110 orders, 197 families). Selected genome assemblies were downloaded from NCBI database.

ANI analyses were made with EzAAI calculator [28] in a Linux environment. POCP calculations were made using the Bio-py POCP-matrix tool in a Linux environment [17, 71]. AAI and POCP comparisons were made in R 4.2.2[82] using the following packages: dplyr 1.1.2, tidyr 1.3.0,

vioplot 0.4.0, ggplot2 3.4.2, stringr 1.5.0, car 3.1-2 and xlsx 0.6.5 [83–89]. Raw outputs from POCP and AAI analysis, the script to generate all tables, statistics and figures and the accession codes of all the genomes used can be found in https://github.com/RiescoR/POCP-VS-AAI.

References

17. **Qin QL, Xie B Bin, Zhang XY, Chen XL, Zhou BC, *et al.*** A proposed genus boundary for the prokaryotes based on genomic insights. *J Bacteriol* 2014;196:2210–2215. doi: 10.1128/JB.01688-14.

28. **Kim D, Park S, Chun J**. Introducing EzAAI: a pipeline for high throughput calculations of prokaryotic average amino acid identity. *Journal of Microbiology* 2021;59:476–480. doi: 10.1007/s12275-021-1154-0.

71. **Lin H**. SilentGene/Bio-py: Bio-py v1.0. 2021;0. doi: 10.5281/zenodo.4954426.

82. **R Core Team**. R: A Language and Environment for Statistical Computing. https://www.r-project.org/ (2022).

83. **Wickham H, François R, Henry L, Müller K, Vaughan D**. dplyr: A Grammar of Data Manipulation, 2023. https://CRAN.R-project.org/package=dplyr.

84. **Wickham H, Vaughan D, Girlich M**. tidyr: Tidy Messy Data, 2023. https://CRAN.R-project.org/package=tidyr.

85. **Adler D, Kelly ST, Elliott T, Adamson J**. vioplot: violin plot, 2022. https://github.com/TomKellyGenetics/vioplot.

86. **Wickham H**. *ggplot2*. Cham: Springer International Publishing; 2016. doi: 10.1007/978-3-319-24277-4.

87. **Wickham H**. stringr: Simple, Consistent Wrappers for Common String Operations, 2023. https://CRAN.R-project.org/package=stringr.

88. **Fox J, Weisberg S**. *An {R} Companion to Applied Regression*. Third. Thousand Oaks {CA}: Sage; 2019.

89. **Dragulescu A, Arendt C**. xlsx: Read, Write, Format Excel 2007 and Excel 97/2000/XP/2003 Files, 2020. https://CRAN.R-project.org/package=xlsx.
